# 

**Published:** 1834-04-01

**Authors:** 


### CONTENTS

OF THE

### MEDICO-CHIRURGICAL REVIEW,

No. XL. APRIL 1, 1834.

### REVIEWS.

I.

RECENT WORKS ON PATHOLOGICAL ANATOMY.

I. Dr. CARSWELL's Illustrations of the Elementary Forms of Diseases.. .. .. 290
II. Principles and Illustrations of Morbid Anatomy. By Dr. HOPE .. .. .. 296
III. CRUVEILHIER's Anatomie Pathologique du Corps Humain, &c... .. .. .. 303
IV. Anatomical Drawings, selected from the Collection of Morbid Anatomy in the Army Medical Museum at Chatham .. .. .. .. .. .. .. .. .. .. 304

II.

Mr. KIERNAN on the Anatomy and Physiology of the Liver .. .. .. .. .. 305

III.

Causes of Decline in the University of Edinburgh .. .. .. .. .. .. .. 315

IV.

Mr. ROGERSON's Jacksonian Prize—Dissertation on Inflammation of the Membranes .. .. .. .. .. .. .. .. .. .. .. .. .. .. .. 320

An Essay on Inflammation. By PHILIP LOVELL PHILLIPS, M.D. .. .. .. .. 324

Mr. ATKINSON's Medical Bibliography .. .. .. .. .. .. .. .. .. .. .. 329

ANATOMICAL PLATES.

I. Mr. SWAN's Plates of the Nerves of the Human Body. Part IV. .. .. .. 334
II. A Series of Anatomical Plates. By JONES QUAIN, M.D. .. .. .. .. .. 335
III. Illustrations of all the most celebrated Medical and Surgical Works .. .. 336
IV. The Anatomy of Inguinal and Femoral Hernia. By E. W. TUSON, F.L.S. .. 336

V.

M. PIORRY on Exploration of the Thoracic and Abdominal Organs, by Means of Percussion .. .. .. .. .. .. .. .. .. .. .. .. .. .. .. 337

VI.

Mr. MACILWAIN on Porrigo .. .. .. .. .. .. .. .. .. .. .. .. .. .. 348

VII.

Transactions of the Medical and Physical Society of Calcutta.

I. On the Climate of Bangalore .. .. .. .. .. .. .. .. .. .. .. 362

II. Medical Topography of Gowhattee.. .. .. .. .. .. .. .. .. .. 336

III. Epidemic Influenza .. .. .. .. .. .. .. .. .. .. .. .. .. 364

IV. On Hepatic Abscesses .. .. .. .. .. .. .. .. .. .. .. .. 365

VIII.

Observations on the original Malformations and total Want of Eyes. By M. VON SEILER .. .. .. .. .. .. .. .. .. .. .. .. .. .. .. 370

IX.

Considerations on Alterations of the Blood in different Diseases. By M. ROCHE .. 381

X.

FACTORY COMMISSION.

I. Instructions from the Central Board.
II. Report of the Medical Commissions } .. .. .. .. .. .. .. .. .. .. 90

XI.

Elements of Materia Medica. By Dr. THOMSON .. .. .. .. .. .. .. .. 403

XII.

An Investigation into the Medicinal Effects of Veratria. By Dr. TURNBULL.. .. 405

XIII.

Traité de Vaccine, sur la Demande du Gouvernement Français .. .. .. .. 417

XIV.

Sir Charles Scudamore on Inhalation in Tubercular Phthisis .. .. .. .. .. .. 420

XV.

A Treatise on Diseases and Injuries of the Nerves. By JOS. SWAN .. .. .. .. .. 423

### PERISCOPE.

PART I.

### Spirit of the English Periodicals, and Notices of English Medical Literature.

1. Suggestions as to the Nature and Treatment of Cerebral Diseases. By Dr. Paine .. .. .. .. .. .. .. .. .. .. .. .. .. .. .. .. .. .. .. 433
2. Dr. Beatty on the Secale Cornutum .. .. .. .. .. .. .. .. .. .. .. 438
3. How to render Sulphate of Magnesia palatable .. .. .. .. .. .. .. .. .. 438
4. Conversion of Lung into Encephaloid Matter .. .. .. .. .. .. .. .. .. 439
5. Dr. Cumming on Hæmatemesis .. .. .. .. .. .. .. .. .. .. .. .. .. 441
6. Dr. Graves on Secretion of Air in Thorax and Abdomen, with Cases.. .. .. 442
7. On the Anterior Membrane of the Eyelid. By Dr. Wallace .. .. .. .. .. 446
8. Dr. Beatty on the Prevention of Uterine Hæmorrhage .. .. .. .. .. 446
9. Dr. Beatty on Mammary Abscess .. .. .. .. .. .. .. .. .. .. .. .. .. 448
10. Dr. Alexander on Vermination .. .... .. .. .. .. .. .. .. .. .. .. 449
11. Dr. Negri on Secale Cornutum .. .. .. .. .. .. .. .. .. .. .. .. .. 450
12. Dr. Beauchamp on Tart. Ant. in Porrigo.. .. .. .. .. .. .. .. .. .. 453
13. Medical Problems. By Dr. Griffin.. .. .. .. .. .. .. .. .. .. .. .. 454
Connexion of Jaundice with Cerebral Affection .. .. .. .. .. .. .. .. 454
14. Dr. Graves on the Anti-Ptyalismal Effect of Iodine .. .. .. .. .. .. .. 455
15. Choleraphobia and Cholera Ecclesiastes.. .. .. .. .. .. .. .. .. .. .. 456
16. The PRECEPTOR—Dr. Stokes on Delirium Tremens and Dyspepsia .. .. .. 457
17. Mr. Middlemore on a peculiar Ulceration of the Eyelid .. .. .. .. .. .. 461
18. Dr. Hunter Lane on Mammary Hypertrophy .. .. .. .. .. .. .. .. .. 464
19. Dr. Graves' Notice of Modern German Books .. .. .. .. .. .. .. .. 467
Effects of Iodine, with Strictures .. .. .. .. .. .. .. .. .. .. .. .. 468

20. Dr. Chester on Fractures of the Lower Jaw .. .. .. .. .. .. .. .. .. 469

21. Tooth lodged in the Right Bronchus .. .. .. .. .. .. .. .. .. .. 471

Additional Cases, by Dr. Evanson .. .. .. .. .. .. .. .. .. .. .. 473

22. Human Teeth the Nuclei of Urinary Calculi.. .. .. .. .. .. .. .. .. 475

23. Mr. Wardrop on the Injurious Effects of Bleeding, with Cases and Remarks.. 476

24. Dr. Aldis on Connexion of Cerebral Disease with Jaundice .. .. .. .. 479

25. Flavour and Odour .. .. .. .. .. .. .. .. .. .. .. .. .. .. .. 480

### PART II.

### Spirit of the Foreign Periodicals.

1. Dr. Ascherson on Vices of Formation .. .. .. .. .. .. .. .. .. .. .. 481
2. Ague treated with the Salicine .. .. .. .. .. .. .. .. .. .. .. .. 482
3. Poison of Sheep-rot communicated to Man .. .. .. .. .. .. .. .. .. 484
4. Sulphate of Cadmium in Specks of Cornea .. .. .. .. .. .. .. .. .. 485
5. Concentrated Laurel-Water in Neuralgia .. .. .. .. .. .. .. .. .. 486
6. Development of the Thymus in Man and Animals .. .. .. .. .. .. .. 487
7. Impediments to Parturition .. .. .. .. .. .. .. .. .. .. .. .. .. 489
8. Torsion of Arteries in Man. By Clot Bey .. .. .. .. .. .. .. .. .. 491
9. Plaster-Moulds for Fractures .. .. .. .. .. .. .. .. .. .. .. .. .. 492
10. Progress of Physiology in the present Century .. .. .. .. .. .. .. 493
11. On the Diagnosis of Cardiac Affections.. .. .. .. .. .. .. .. .. .. 494
12. Medical Properties of Creosote .. .. .. .. .. .. .. .. .. .. .. .. 497
13. Anatomical Notanda—(Anatomy of the Eye) .. .. .. .. .. .. .. .. 498
On the Ganglion Oticum .. .. .. .. .. .. .. .. .. .. .. .. .. .. .. 498
14. The last Illness of Goethe.. .. .. .. .. .. .. .. .. .. .. .. .. .. 499
15. Calcareous Incrustations on the Crystalline Lens.. .. .. .. .. .. .. 501
16. Muller's Experiments on the Blood .. .. .. .. .. .. .. .. .. .. .. 503

### Institute of France.

17. Induction of Premature Labour .. .. .. .. .. .. .. .. .. .. .. .. 503
18. Cow-pox, Origin of .. .. .. .. .. .. .. .. .. .. .. .. .. .. .. .. 504
19. Petit on the Treatment of Cholera.. .. .. .. .. .. .. .. .. .. .. .. 505
20. Nutrition and Diseases of the Humour of the Eye .. .. .. .. .. .. .. 505
21. Suture of the Perineum .. .. .. .. .. .. .. .. .. .. .. .. .. .. .. 506
22. Epizootics .. .. .. .. .. .. .. .. .. .. .. .. .. .. .. .. .. .. .. 506
23. Medical Responsibility .. .. .. .. .. .. .. .. .. .. .. .. .. .. .. 507

### Academy of Sciences.

24. On Chromate of Potash .. .. .. .. .. .. .. .. .. .. .. .. .. .. .. 507
25. Causes of Proportion of the Sexes .. .. .. .. .. .. .. .. .. .. .. 508
26. Development of the Embryo in the Mammiferi .. .. .. .. .. .. .. .. 508

27. Lacerated Perineum, Treatment of.. .. .. .. .. .. .. .. .. .. .. .. 510
28. Professor Bouillaud's opinion respecting Typhus Fever .. .. .. .. .. 514
29. Hearing through the Apertures made by the Trephine .. .. .. .. .. .. 516
30. Purification of Theatres of Dissection .. .. .. .. .. .. .. .. .. .. 516
31. On Adulterations of Fecula, &c. .. .. .. .. .. .. .. .. .. .. .. .. 516
32. Encephaloid Disease of the Kidney .. .. .. .. .. .. .. .. .. .. .. 517

33. Treatment of Encysted Tumor in the Larynx .. .. .. .. .. .. .. .. .. 519
34. Evils and Treatment of Flat-footedness.. .. .. .. .. .. .. .. .. .. .. 520
35. Dislocation of the Astragalus .. .. .. .. .. .. .. .. .. .. .. .. .. 520
36. Spina Ventosa of the Hand .. .. .. .. .. .. .. .. .. .. .. .. .. 523
37. M. Bouillaud on Follicular Enteritis .. .. .. .. .. .. .. .. .. .. .. 524
38. Extirpation of Fungoid Tumor of Dura Mater .. .. .. .. .. .. .. .. .. 526

PART III.

### Clinical Review and Hospital Reports.

1. Remarks on some frequent Forms of Venereal Ulcer, by Mr. H. J. Johnson .. 529
2. Report of Cases treated by Mr. Bower, at the Rochdale General Dispensary .. 543
3. Lancaster Lunatic Asylum .. .. .. .. .. .. .. .. .. .. .. .. .. .. 546
4. Mr. Guthrie on Stricture .. .. .. .. .. .. .. .. .. .. .. .. .. .. 548
5. M. Bland on the Fibrinous Polypi in the Cavities of the Heart. (Hôpital de Beaucaire) .. .. .. .. .. .. .. .. .. .. .. .. .. .. .. .. 551
6. Traumatic Tetanus .. .. .. .. .. .. .. .. .. .. .. .. .. .. .. .. 555
7. Neuralgic Affections of Stumps. (St. Bartholomew's).. .. .. .. .. .. .. 555
8. Mr. B. Cooper's Clinical Remarks on Injuries of the Head. (Guy's Hospital).. 556
9. Medical Reform .. .. .. .. .. .. .. .. .. .. .. .. .. .. .. .. 560

### Miscellanies.

FOREIGN MEDICAL POLITICS.

1. Report of the Parisian Academy of Medicine .. .. .. .. .. .. .. .. .. 561
2. Prussian System of Medical Polity .. .. .. .. .. .. .. .. .. .. .. .. 563
3. Medical Statistics and Reform.. .. .. .. .. .. .. .. .. .. .. .. .. .. 567
4. Sketch of a Plan of Practicable Medical Reform in this Country .. .. .. 569
5. Northern Provincial Meeting in Ireland.. .. .. .. .. .. .. .. .. .. .. 571
6. Obituary—Sir W. Franklin .. .. .. .. .. .. .. .. .. .. .. .. .. .. 572
7. Bibliographical Record .. .. .. .. .. .. .. .. .. .. .. .. .. .. .. 573

EXTRA-LIMITES.

1. Dr. Wise's reply to Mr. Arnott .. .. .. .. .. .. .. .. .. .. .. .. .. 577

